# Association between cumulative average triglyceride glucose-body mass index and the risk of CKD onset

**DOI:** 10.3389/fendo.2025.1525078

**Published:** 2025-03-31

**Authors:** Yu Wang, Bin Chen, Chongsen Zang, Jie Hou

**Affiliations:** Department of Nephrology, the First Hospital of Jilin University, Changchun, Jilin, China

**Keywords:** CHARLS, chronic kidney disease, triglyceride glucose-body mass index, old age, cumulative change

## Abstract

**Background:**

Chronic kidney disease (CKD) has become a significant global public health challenge, which was reported to be highly correlated with the triglyceride glucose-body mass index (TyG-BMI). Nevertheless, literature exploring the association between changes in the TyG-BMI and CKD incidence is scant, with most studies focusing on individual values of the TyG-BMI. We aimed to investigate whether cumulative average in the TyG-BMI were associated with CKD incidence.

**Methods:**

Data in our study were obtained from the China Health and Retirement Longitudinal Study (CHARLS), which is an ongoing nationally representative prospective cohort study. The exposure was the cumulative average TyG-BMI from 2011 to 2015. The TyG-BMI was calculated by the formula ln [TG (mg/dl) × FBG (mg/dl)/2] × BMI (kg/m^2^), and the cumulative average TyG-BMI was calculated as follows: (TyG-BMI_2011_+ TyG-BMI_2015_)/2. Logistic regressions were used to determine the association between different quartiles of cumulative average TyG-BMI and CKD incidence. Meanwhile, restricted cubic spline was applied to examine the potential nonlinear association of the cumulative average TyG-BMI and CKD incidence. In addition, subgroup analysis was used to test the robustness of results.

**Results:**

Of the 6117 participants (mean [SD] age at baseline, 58.64 [8.61] years), 2793 (45.7%) were men. During the 4 years of follow-up, 470 (7.7%) incident CKD cases were identified. After adjusting for potential confounders, compared to the participants in the lowest quartile of cumulative average TyG-BMI, participants in the 3rd and 4th quartile had a higher risk of CKD onset. The ORs and 95%CIs were [1.509(1.147, 1.990)] and [1.452(1.085, 1.948)] respectively. In addition, restricted cubic spline showed the cumulative average TyG-BMI had a liner association (*p*-nonlinear = 0.139).

**Conclusions:**

The cumulative average in the TyG-BMI was independently associated with the risk of CKD in middle-aged and older adults. Monitoring long-term changes in the TyG-BMI may assist with the early identification of individuals at high risk of CKD.

## Introduction

Chronic kidney disease (CKD), one of the major non-communicable chronic diseases, is a leading cause of morbidity and mortality of the population all over the world. There are more than 10% of the population suffers from CKD and most of them are elderly (1). In 2017, the number of persons who died because of CKD accounted for 2.2% of all deaths (2). It is estimated that CKD may rank the fifth cause of years of life lost in 2040 (3). In addition, chronic kidney disease is asymptomatic in the early stage, and some patients have already entered the terminal stage and are often accompanied by some complication when they are recognized such as Osteoporosis, cardiovascular disease and so on (4–6). This not only seriously affects the physical and mental health of patients, but also brings a huge economic burden to families and society. Therefore, exploring suitable predictors of CKD can improve the identification of people at high risk and diagnose patients with CKD earlier. It is essential for preventing or delaying CKD and its complications, avoiding premature death of patients and improving quality of life.

Insulin resistance (IR) is a decrease in the sensitivity of cells to recognize insulin, resulting in the body’s inability to absorb and utilize glucose in a timely manner. Many studies showed that IR played a key role in the onset and progression of CKD (7–9). Although there are several methods for IR measurement, they are not widely used in clinical practice due to time and money consuming (10, 11). Triglyceride glucose-body mass index (Tyg-BMI), an indicator based on triglyceride (TG), fasting blood glucose (FBG) and BMI and has been proved to be strongly associated with hypertension, diabetes, and stroke (12–14). Compared with a single indicator, Tyg-BMI combined information on lipids, glucose, and obesity, was able to better reflect IR, and was considered a valid and reliable alternative to IR (15). Several previous cross-sectional studies suggested that TyG-BMI was associated with CKD, but they focused on a single measurement of TyG-BMI and neglected the long-term effects of TyG-BMI on CKD (16, 17).

TG, FBG and BMI tend to be influenced by various factors such as diet and anxiety, which in turn can lead to fluctuations and changes in TyG-BMI over time (18, 19). Previous studies have shown that cumulative TyG-BMI was closely associated with cardiovascular disease onset including stroke, heart attack, hypertension and so on (20–22). Although previous studies have provided valuable insights, the association between longitudinal cumulative average TyG-BMI exposure and the development of CKD is unclear. Therefore, we aimed to explore the association between longitudinal cumulative average TyG-BMI and CKD in the Chinese to provide theoretical evidence for the screening and prevention of CKD at an early stage.

## Methods

### Study population

All data in this study was sourced from the China Health and Retirement Longitudinal Study (CHARLS), a nationally representative cohort study conducted on people aged ≥45 years in China. This study started a baseline survey in 2011, followed by follow-up surveys every two years, and four rounds of follow-up surveys have been completed up to now. Given that information on blood samples was collected only in 2011 and 2015 surveys, data from 2011 to 2015 was selected for analysis in this study.

**Figure 1:**
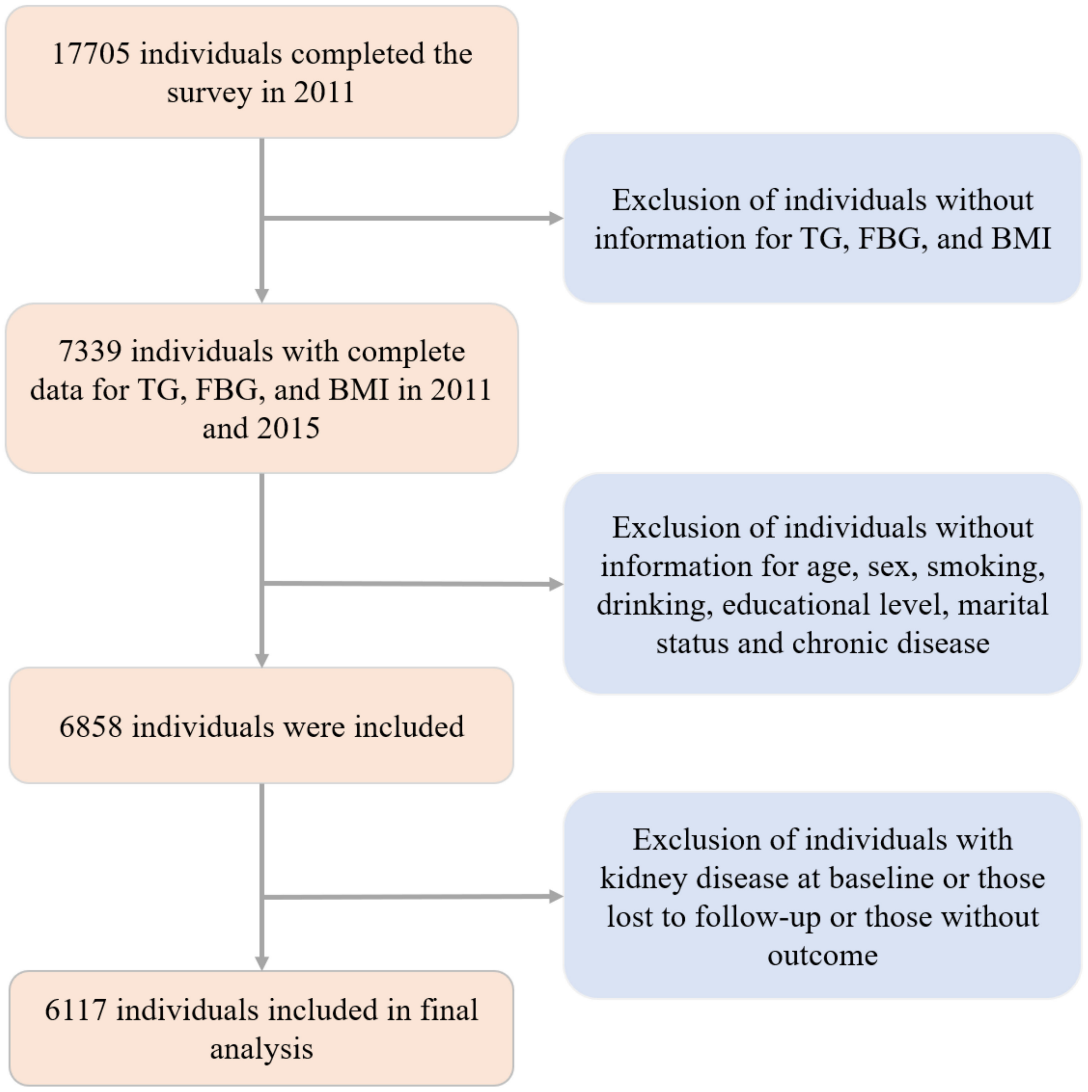
Flowchart of the subjects in the study.

We first included 17705 participants at wave 1, and we excluded those without information for TG, FBG, and BMI at wave 1 and wave 3, those with CKD at baseline or those lost to follow-up or without outcome information, and those without covariate. Ultimately, a total of 6117 eligible participants were included in our analysis (**Figure 1**).

### Assessment of the cumulative average TyG-BMI

The TyG-BMI was calculated by the formula ln [TG (mg/dl) × FBG (mg/dl)/2] × BMI (kg/m^2^). BMI was calculated using height and weight of participants. The cumulative average TyG-BMI was calculated using the following formula: (TyG-BMI_2011_ + TyG-BMI_2015_)/2

### Assessment of incident CKD events

The primary outcome of this study is chronic kidney disease. In each survey of CHARLS, participants were asked “Have you been diagnosed by a doctor with kidney disease (except for tumor or cancer) (23)?” Participants who self-reported “yes” were defined as CVD patients. Participants self-reported not being diagnosed with CKD by a doctor in 2011 and self-reported being diagnosed with CKD by a doctor at subsequent follow-up were identified as having a CKD event.

### Covariates

This study collected covariates at baseline including age, sex (men or women), smoking status (yes or no), drinking status (yes or no), educational level (primary school or below or secondary school or above), marital status (married or others) and chronic disease (yes or no).

Chronic disease included hypertension, diabetes, dyslipidemia, and heart attack. Participants who self-reported having been diagnosed with hypertension by a physician or had systolic blood pressure ≥ 140 mmHg and (or) diastolic blood pressure ≥ 90 mmHg were considered to have hypertension. Participants who self-reported having been diagnosed with diabetes by a doctor or fasting blood glucose ≥ 7.0 mmol/L or glycosylated hemoglobin ≥ 6.5% were considered to have diabetes. Participants who self-reported having been diagnosed with dyslipidemia or heart attack by a doctor were considered to have dyslipidemia or heart attack.

### Statistical analysis

All study participants were classified in four groups based on quartiles of the cumulative average TyG-BMI [from Quartile 1 (Q1) to Quartile 4 (Q4)]. Baseline characteristics of study participants were described using mean ± standard deviation (SD) or numbers (percentage). one-way analysis of variance (ANOVA) or Pearson chi-square test were used for comparison between different groups. Logistic regressions were used to determine the association between different quartiles of cumulative average TyG-BMI and CKD incidence. We built three models to calculate and report odds ratios (OR) of the outcomes, with the corresponding 95% confidence interval (CI). Model I did not include any covariables. Model II adjusted for age and sex. Model III adjusted for all covariables (age, sex, smoking status, drinking status, educational level, marital status and chronic diseases). Meanwhile, restricted cubic spline (RCS) was applied to examine the potential nonlinear association of the cumulative average TyG-BMI and CKD incidence. In addition, subgroup analysis and interaction analysis were used to detect the potential modifications.

We used R version 4.3.1 (R Foundation for Statistical Computing) for all statistical analyses. A two-tailed p < 0.05 was considered statistically significant.

## Results

### Baseline characteristics of study participants

**Table 1** showed the baseline characteristics of all participants. The average age of the participants was 58.64 years old, and 2793(45.7%) of them were men. **Table 2** showed the baseline characteristics of the participants in different groups classified by quartiles of cumulative average TyG-BMI. Compared with participants in the lowest quartile group (Q1), those in groups with higher levels of the cumulative average TyG-BMI (Q2-Q4) were more likely to be younger, women and married person, showed a higher educational level and had a greater frequency of hypertension, dyslipidemia, diabetes and heart disease (*p* < 0.05).

**Table 1:** Baseline characteristics of participants in the study.

	Mean ± SD/n (%)
Age	58.64 ± 8.61
Sex	
Men	2793 (45.7)
Women	3324 (54.3)
BMI (kg/m^2^)	23.90 ± 3.99
Smoking	
Yes	2337 (38.2)
No	3780 (61.8)
Drinking	
Yes	2044 (33.4)
No	4073 (66.6)
Educational level	
Primary school or below	4280 (70.0)
Secondary school or above	1837 (30.0)
Marital status	
Married	5225 (85.4)
Others	892 (14.6)
Hypertension	
Yes	3507 (57.3)
No	2610 (42.7)
Diabetes	
Yes	1069 (17.5)
No	5048 (82.5)
Dyslipidemia	
Yes	592 (9.7)
No	5525 (90.3)
Heart disease	
Yes	694 (11.3)
No	5423 (88.7)
Chronic disease	
Yes	3425 (56.0)
No	2692 (44.0)

**Table 2:** Baseline characteristics of participants classified by quartiles of the cumulative average TyG-BMI.

	Quartile 1	Quartile 2	Quartile 3	Quartile 4	P
**Age**	61.14 ± 9.31	58.93 ± 8.27	57.80 ± 8.33	56.70 ± 8.61	<0.001
**Sex**					<0.001
Men	885 (57.9)	721 (47.2)	641 (41.9)	546 (35.7)	
Women	644 (42.1)	808 (52.8)	889 (58.1)	983 (64.3)	
**BMI (kg/m^2^)**	19.59 ± 1.56	22.58 ± 1.25	24.85 ± 1.45	28.56 ± 3.85	<0.001
**Smoking**					<0.001
Yes	784 (51.3)	578 (37.8)	538 (35.2)	437 (28.6)	
No	745 (48.7)	951 (62.2)	992 (64.8)	1092 (71.4)	
**Drinking**					<0.001
Yes	615 (40.2)	530 (34.7)	488 (31.9)	411 (26.9)	
No	914 (59.8)	999 (65.3)	1042 (68.1)	1118 (73.1)	
**Educational level**					<0.001
Primary school or below	1161 (75.9)	1092 (71.4)	1022 (66.8)	1005 (65.7)	
Secondary school or above	368 (24.1)	437 (28.6)	508 (33.2)	524 (34.2)	
**Marital status**					<0.001
Married	1272 (83.2)	1276 (83.5)	1333 (87.1)	1344 (87.9)	
Others	257 (16.8)	253 (16.5)	197 (12.9)	185 (12.2)	
**Hypertension**					<0.001
Yes	429 (28.1)	568 (37.1)	701 (45.8)	912 (59.6)	
No	1100 (71.9)	961 (62.9)	829 (54.2)	617 (40.4)	
**Diabetes**					<0.001
Yes	153 (10.0)	195 (12.8)	270 (17.6)	451 (29.5)	
No	1376 (90.0)	1334 (87.2)	1260 (82.4)	1078 (70.5)	
**Dyslipidemia**					<0.001
Yes	54 (3.5)	92 (6.0)	160 (10.5)	286 (18.7)	
No	1475 (96.5)	1437 (94.0)	1370 (89.5)	1243 (81.3)	
**Heart disease**					<0.001
Yes	137 (9.0)	146 (9.5)	163 (10.7)	248 (16.2)	
No	1392 (91.0)	1383 (90.5)	1367 (89.3)	1281 (83.8)	
**Chronic disease**					<0.001
Yes	611 (40.0)	760 (49.7)	913 (59.7)	1141 (74.6)	
No	918 (60.0)	769 (50.3)	617 (40.3)	388 (25.4)	

### Association between cumulative average TyG-BMI and CKD

**Figure 2:**
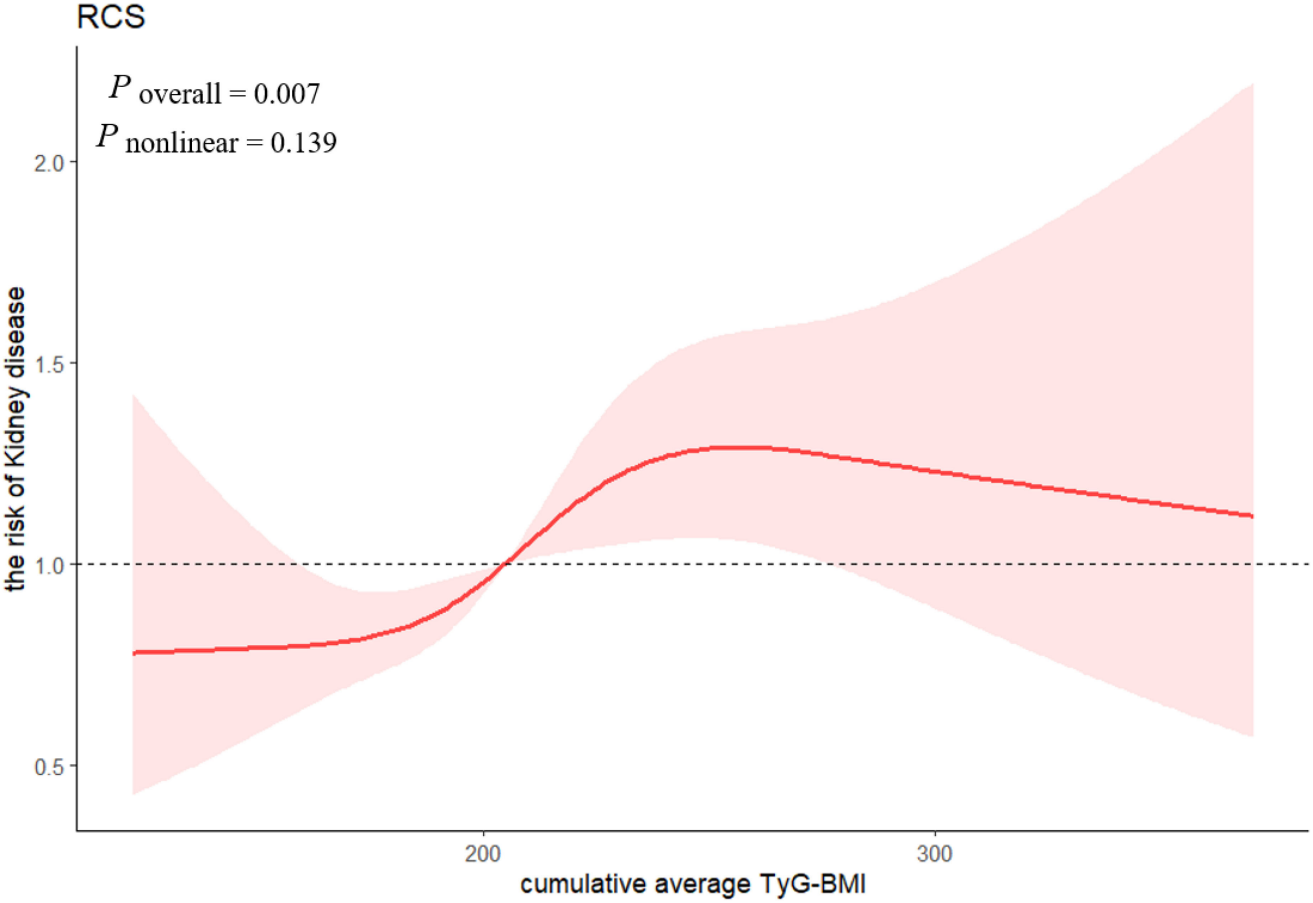
Association between the cumulative average TyG-BMI and Kidney disease.

As shown in **Table 3**, after adjustment for all covariates, participants in the Q3 and Q4 groups had a higher risk of CKD onset compared with those in the Q1 group, ORs and 95%CIs were 1.509(1.147, 1.990) and 1.452(1.085, 1.948) respectively and *p*-value for trend < 0.05. Likewise, when considered as a continuous variable, per 1 SD rise in the cumulative average TyG-BMI was significantly associated with the risk of CKD onset, OR (95%CI) was 2.004(1.236, 3.196). **Figure 2** showed a linear association between the cumulative average TyG-BMI and CKD onset. (*p* for overall = 0.007, *p* for nonlinear = 0.139)

**Table 3:** Association between the cumulative average TyG-BMI and Kidney disease.

Cumulative variables	Model I		Model II		Model III	
	OR (95%CI)	*p*-value	OR (95%CI)	*p*-value	OR (95%CI)	*p*-value
**TyG-BMI (per 1SD)**	2.113 (1.335, 3.297)	0.001	2.157(1.360, 3.374)	<0.001	2.004(1.236, 3.196)	0.004
TyG-BMI						
Q1	1(reference)	***-***	1(reference)	***-***	1(reference)	***-***
Q2	1.049 (0.789, 1.394)	0.741	1.047(0.787, 1.392)	0.751	1.032(0.775, 1.374)	0.829
Q3	1.531(1.170, 2.009)	0.002	1.548(1.182, 2.032)	0.002	1.509(1.147, 1.990)	0.003
Q4	1.499(1.134, 1.984)	0.005	1.514(1.144, 2.008)	0.004	1.452(1.085, 1.948)	0.012
*p*-value for trend	0.119		<0.001		0.002	

Model I did not include any covariables.

Model II adjusted for age and sex.

Model III adjusted for all covariables (age, sex, smoking status, drinking status, educational level, marital status and chronic diseases).

### Results of the subgroup analysis

**Figure 3:**
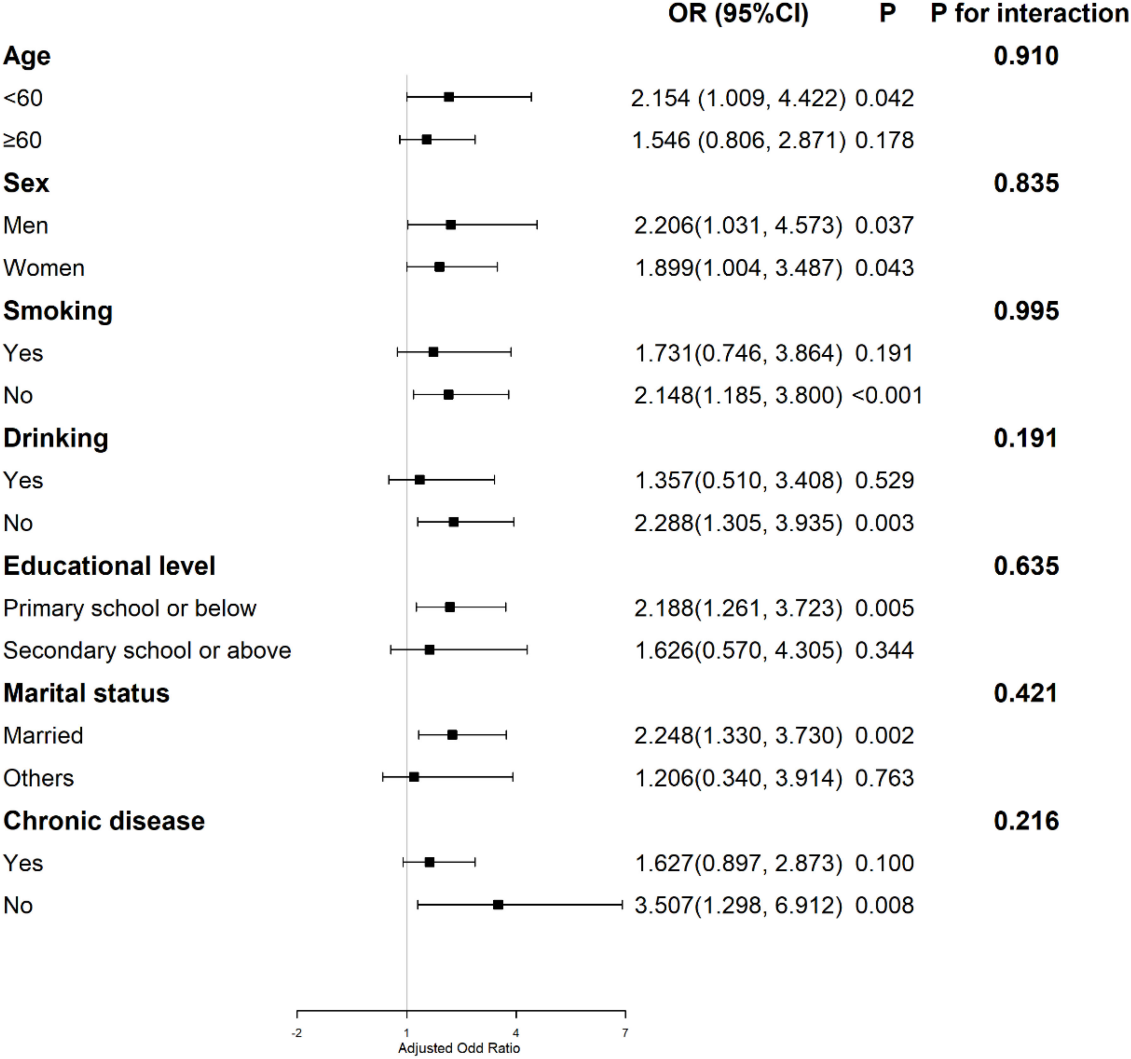
Subgroup analyses of the association between the cumulative average TyG-BMI and Kidney disease.

To further explore the potential modification role of other factors, we conducted interaction analysis and subgroup analysis of cumulative average TyG-BMI and covariates. The results showed that age, sex, smoking, drinking, educational level, marital status and chronic disease did not modify the association between cumulative average TyG-BMI and the risk of CKD onset (*p*-interaction >0.05) (**Figure 3**).

## Discussion

In this study, we explored the association between cumulative average TyG-BMI index and the risk of CKD onset using data from CHARLS 2011-2015. We found that cumulative average TyG-BMI was linearly associated with the risk of CKD onset, and that the higher cumulative average TyG-BMI index was associated with a greater risk of CKD onset even after adjusting for all confounders.

Chronic kidney disease, as one of the major public health problems, has a complex pathogenesis and is closely related to traditional risk factors such as hypertension, diabetes and obesity (24). Patients with chronic kidney disease often lack obvious symptoms at the early stages, so most patients are found to be in later stages with multiple complications, lower quality of life and higher mortality (25). Therefore, it is necessary to find early predictive indicators for chronic kidney disease.

TyG index combined the effects of blood glucose and lipids and has been proposed as a predictor of a variety of chronic diseases, including heart failure, ischemic stroke, coronary artery disease, obstructive sleep apnea, CKD and so on (26–34). Compared with TyG index, TyG-BMI, a new combined indicator that not only considered blood glucose and lipids, but also obesity, could reflect the status of metabolism of individuals more comprehensively. Given that both the measurement and calculation of TyG-BMI were simpler, it has attracted a lot of attention from scholars. Currently, TyG-BMI is considered closely associated with some chronic diseases. A cohort study conducted in Japanese medical examinations population showed that the risk of diabetes in the future was 1.51 times as high for every 1 standard deviation increased in Tyg-BMI after adjusting for confounders (35). According to dang et al, Tyg-BMI was positively associated with total cardiovascular disease, congestive heart failure, myocardial infarction, angina pectoris, and coronary heart disease (36). In addition, the studies of Peng and shao indicated that Tyg-BMI was closely associated with the occurrence of non-alcoholic fatty liver disease and stroke respectively (14, 37). However, as a metabolic disease, there are limited studies focused on the association between Tyg-BMI and CKD. Li et al. investigated the association between Tyg-BMI and estimated glomerular filtration rate (eGFR) and found that Tyg-BMI was negatively correlated with eGFR (16). However, this study only focused on a single measurement and obtained data from only one survey, so it is not possible to determine the temporal order of them. On this basis, we further explored the association between cumulative average mean Tyg-BMI and the risk of chronic kidney disease using longitudinal data. We considered the changes in Tyg-BMI over time and revealed the long-term impact of Tyg-BMI on chronic kidney disease. In addition, the results of our subgroup analysis showed that age, sex, smoking status, drinking status, educational level, marital status and chronic disease did not significantly modify the association between Tyg-BMI and the risk of chronic kidney disease onset, which further emphasized the robustness of our results. This finding suggested people that regular assessment the level of Tyg-BMI is important to help identify individuals at high risk for chronic kidney disease, prevent the kidney disease onset and reduce the burden of disease.

In addition, participants with higher cumulative TyG-BMI in this study were less likely to smoke and drink, which may be related to the fact that they made more positive lifestyle to improve their health after they realized the abnormalities in blood lipids and BMI. In addition, compared with participants in the lowest quartile group, those in groups with higher levels of the cumulative average were more likely to be women and married person, showed a higher educational level, which was closely related to various factors such as women’s physiological factors (such as postmenopausal hormonal changes), life stresses from multiple aspects faced by the participants, and lifestyle choices of participants (38–41). In a word, the change of TyG-BMI was the consequence of combination of factors, and more researches were needed to explore this in the future.

Tyg-BMI may influence the development of chronic kidney disease by the following mechanisms: Firstly, TyG-BMI has been recommended as a marker for the assessment of IR and IR-associated diseases, so the potential mechanism of association between Tyg-BMI and CKD may be related to IR (42). Insulin receptor was widely distributed on the cell membrane of tubular cell and podocyte and played an important role in the process of insulin signaling. The effectiveness of this signaling directly affected various biological processes, such as the synthesis of nitric oxide (NO). NO is an important vasodilator that could increase renal blood flow and is essential for maintaining normal kidney function (43, 44). IR could affect the process of insulin signaling, leading to a decrease in the number of synthesized NO, which further led to a decrease of renal blood flow, impaired podocyte viability and tubular function. In addition, IR could have an impact on the kidney function by triggering an oxidative stress, systemic inflammation or increasing the level of angiotensin II (45–47). Secondly, obesity, as a major component of Tyg-BMI, may played an important role in the association of Tyg-BMI and kidney function. On the one hand, Direct cytotoxic effects of obesity that may directly damage the kidneys. When triglycerides and ceramides were excessive in kidney cells, they directly damaged the structure and function of kidney cells (48, 49). On the other hand, as an endocrine organ, obesity secreted pro-inflammatory factors such as tumor necrosis factor α (TNF-α) and interleukin 6 (IL-6), which caused a long-term inflammatory state and affected the function of the kidney (50). Finally, IR may further aggravate in the state of obesity and have a greater impact on kidney (51). Of course, more studies are needed to explore the mechanism of Tyg-BMI on the kidney in the future.

In a word, our results suggested the significance of using TyG-BMI especially cumulative average TyG-BMI for CKD onset. IR was closely associated with the development of CKD and it could be measured by hyperinsulinemic-euglycemic clamp test and homeostasis model assessment of IR (HOMA-IR) (10, 11). Compared with these methods, TyG-BMI, a marker for the assessment of IR, was calculated based on common laboratory indicators and was a practical and easy-to-use tool for routine clinical practice (42). Early identification of individuals at high risk for CKD can help physicians and individuals to take interventions earlier including lifestyle adjustments and enhanced monitoring, which is important for preventing the occurrence of CKD and reducing the burden of disease. TyG-BMI provided another useful tool for health workers to identify individuals at high risk for CKD. TyG-BMI was even more important in resource-limited settings due to its ease of access and computational simplicity.

The work has the following strengths: Firstly, this study used longitudinal panel data to investigate the association between Tyg-BMI and kidney, rather than collecting data in the same survey, which enhanced the reliability of causal association. Secondly, we considered the effect of time on Tyg-BMI, so that we not only could obtain a more stable measurement, but also observe the long-term effect of Tyg-BMI on the kidney, which was closer with the real condition. However, this study has some limitations. Firstly, the definition of CKD in this study was based on self-reported by participants similar with other studies. Although this definition had been used in previous studies, we also acknowledged it may increase the risk of bias, which may have resulted in a lower number of patients, thus underestimating the effect of Tyg-BMI on the kidney (23, 52, 53). Secondly, although we have considered many possible confounding factors based on previous literature, there may be other factors that may affect the association between them were not considered by us due to the limitations of the database. Thirdly,

considering that incomplete data may affect the validity of the analysis and the reliability of the results to some extent, we excluded participants with incomplete data. But we acknowledged that this may affect the representativeness of the sample. So, association between cumulative average TyG-BMI and CKD may need to be further explored in a more representative sample in the future. Finally, this study was conducted in Chinese, so caution was needed when generalizing the conclusions to other non-Chinese populations.

## Conclusions

In this study, we revealed that cumulative average TyG-BMI was positively associated with the risk of CKD onset in participants aged 45 years and above of Chinese. Consequently, monitoring long-term changes in the TyG-BMI should prioritize CKD prevention strategies.

## Data Availability

The original contributions presented in the study are included in the article/supplementary material. Further inquiries can be directed to the corresponding author/s.
